# Anti-Proliferative Effects of an Extra-Virgin Olive Oil Extract Enriched in Ligstroside Aglycone and Oleocanthal on Human Liver Cancer Cell Lines

**DOI:** 10.3390/cancers11111640

**Published:** 2019-10-24

**Authors:** Daniela De Stefanis, Salvatore Scimè, Simone Accomazzo, Andrea Catti, Andrea Occhipinti, Cinzia Margherita Bertea, Paola Costelli

**Affiliations:** 1 Department of Clinical and Biological Sciences, Experimental Medicine and Clinical Pathology Unit, University of Turin, 10125 Torino, Italy; daniela.destefanis@unito.it (D.D.S.); scime.salvatore@gmail.com (S.S.); simone.accomazzo@gmail.com (S.A.); andrea.catti@tiscali.it (A.C.); 2 Department of Life Sciences and Systems Biology, University of Turin, 10125 Torino, Italy; cinzia.bertea@unito.it (C.M.B.); a.occhipinti@abelnutraceuticals.com (A.O.)

**Keywords:** cell death, cell proliferation, oxidative stress, TNF α, autophagy

## Abstract

Oleocanthal and ligstroside aglycone are olive oil-derived polyphenols. The former interferes with tumor growth with minor or no cytotoxicity on non-tumorigenic primary cell lines. The information about the bioactivity of ligstroside aglycone are scanty, with the exception of a known antioxidant power. Hepatocellular carcinoma is a malignant tumor with high mortality rates. Systemic chemotherapy is only marginally effective and is frequently complicated by toxicity. Previous observations have shown that hepatocellular carcinoma cell lines become more sensitive to taxol when it is combined with Tumor Necrosis Factor α (TNFα). The present work aimed to assess the effects of a polyphenolic extract containing both oleocanthal and ligstroside aglycone on proliferation and/or death in three liver cancer cell lines (HepG2, Huh7 and Hep3B). The possibility to enhance such effect by the addition of TNFα was also investigated. Both cell proliferation and death were enhanced by the exposure to the polyphenolic extract. Such effect was associated with induction of autophagy and could be potentiated by TNFα. The presence of ligstroside aglycone in the extract lowered the oleocanthal concentration required for cytotoxicity. These results show for the first time that the effects of a polyphenol extract can be potentiated by TNFα and that modulation of autophagy likely account for these effects.

## 1. Introduction

A high dietary intake of olive oil is associated with reduced incidence of cardiovascular and metabolic diseases, Alzheimer disease, osteoporosis and cancer [1,2,3]. Such protection was associated with the several biological activities exerted by olive oil and its phenolic compounds, including antioxidant, anti-inflammatory, antitumor and antidiabetic properties [4,5,6].

Olive-derived polyphenols in olive oil include tyrosol, hydroxytyrosol, oleocanthal (OC), oleacein, ligstroside aglycone (LA) and oleuropein [7]. Among these, OC represents only 10% of the total amount of phenolic compounds [8], although its concentration depends on several variables such as olive cultivar, environmental factors, extraction and storage conditions [5]. Despite its low concentration, OC is gaining growing interest due to its bioactivity. Another relevant polyphenol in extra-virgin olive oil (EVOO) is LA, from which OC derives. While the information on LA bioactivity are scanty, a few years ago it was demonstrated to behave as an antioxidant [9].

Among the several bioactivities of OC, the ability to interfere with tumor growth is gaining increasing interest, further stimulated by the observation that it appears to selectively affect cancer cells, with minor or no cytotoxicity on non-tumorigenic primary cell lines [10,11,12]. The effects of OC were tested on tumor cell lines of different origins [13,14], showing that this polyphenol interferes with molecular processes involved in both cell proliferation and death, resulting in reduced tumor growth.

Hepatocellular carcinoma (HCC) is the fifth most common malignancy worldwide with high morbidity and mortality rates [15], affecting approximately one million individuals/year, the incidence being equal to the mortality rate. Systemic chemotherapy is only marginally effective and is frequently complicated by toxicity that is not faced by survival benefit. Agents such as paclitaxel, tamoxifen, octreotide or antiandrogens are completely ineffective [16]. In addition, chemoresistance frequently occurs, being one of the main problems in the management of HCC patients. Along this line, there is an active search for agents that could be coupled to the standard chemotherapy treatment in order to allow a reduction of drug dosage, resulting in reduced toxicity as well as delayed resistance onset. In this regard, previous observations have shown that HCC cell lines became more sensitive to taxol if they were exposed to the drug in the presence of Tumor Necrosis Factor α (TNFα) [17].

At present, only a few data describe the effects of OC on human HCC. Indeed, OC has been shown to inhibit HCC growth and metastasis through mechanisms involving, at least, reactive oxygen species (ROS) production and signal transducer and activator of transcription 3 (STAT3) activation [10,11]. By contrast, no data are available in the literature for LA.

To fill this gap, the present work aimed to assess the ability of a polyphenolic extract containing both LA and OC to affect proliferation and/or death in the liver cancer cell lines HepG2, Huh7 and Hep3B, and if such effect could be enhanced by the addition of TNFα.

## 2. Results

### 2.1. Characteristics of the Extra-Virgin Olive Oil (EVOO) Phenolic Extract

Chromatographic separation of the olive oil polyphenolic extract by reverse phase HPLC–DAD-ESI-MS/MS revealed the prevalent presence of two compounds (OC and LA) and the presence only in traces of the other two, as shown in Table 1. The compounds were identified by comparison of fragmentation spectra with literature data and quantified by an external calibration curve with chlorogenic acid (Table 1).

### 2.2. Effect of the Phenolic Extract on Hepatocellular Carcinoma (HCC) Cell Proliferation and Death

**Figure 1:**
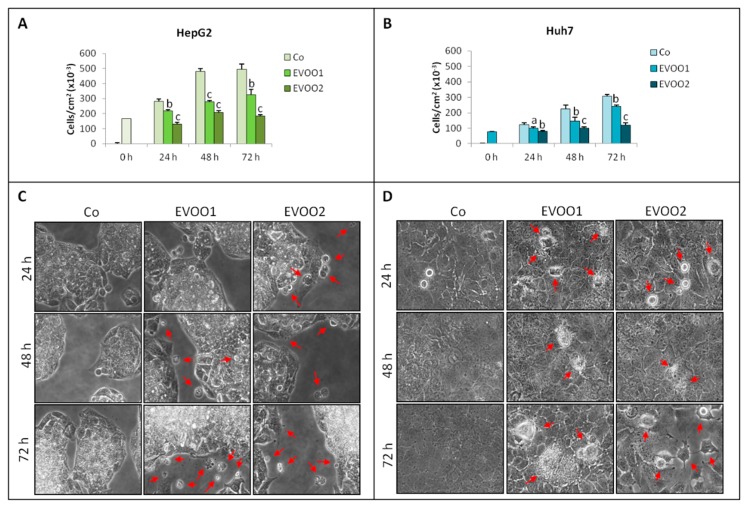
Phenolic extract reduces liver cancer cell proliferation. (**A**,**C**) Growth curve and morphological analysis on HepG2 cell line. (**B**,**D**) Growth curve and morphological analysis on Huh7 cell line. The cells were incubated for 24–72 h with two different doses of the extra-virgin olive oil (EVOO) extract. The results are representative of three experiments. Data are presented as mean ± standard deviation (SD). (a: *p* < 0.05, b: *p* < 0.01, c: < 0.001 versus Control). Red arrows indicate vacuolated or condensed cells and debris that could not be observed in controls and that recall death by apoptosis.

Liver cancer cells (HepG2, Huh7 and Hep3B) were incubated for 24–72 h with two different doses of the phenolic extract: 4.81 µg/mL (EVOO1), corresponding to 5 µM OC and 9 µM LA, and 9.62 µg/mL (EVOO2), containing 10 µM OC and 18 µM LA. The results show that the cell number was reduced by the phenolic extract in a dose- and time-dependent manner (Figure 1A,B and Supplementary Figure S1A). In particular, in HepG2 cells, EVOO1 induced a persistent reduction of cell numbers already evident at 24 h treatment. The pattern was even worse in HepG2 cells exposed to EVOO2, with cell number remaining comparable to time 0 (Figure 1A). Qualitatively speaking, cell number was reduced in the Huh7 cells similarly to HepG2 cultures, although the effect of EVOO1 appeared less marked in the former than in the latter (Figure 1B).

The morphological analysis shows a picture that is consistent with the growth curve for both cell lines (Figure 1C,D and Supplementary Figure S1B). Both extract doses caused the appearance of vacuolated or condensed cells and debris that could not be observed in controls and that recall death by apoptosis. Moreover, HepG2 cells exposed to EVOO2 during 72 h did not reproduce the typical multilayer growth (Figure 1C), while in Huh7 cultures, large empty spaces could be observed (Figure 1D).

**Figure 2:**
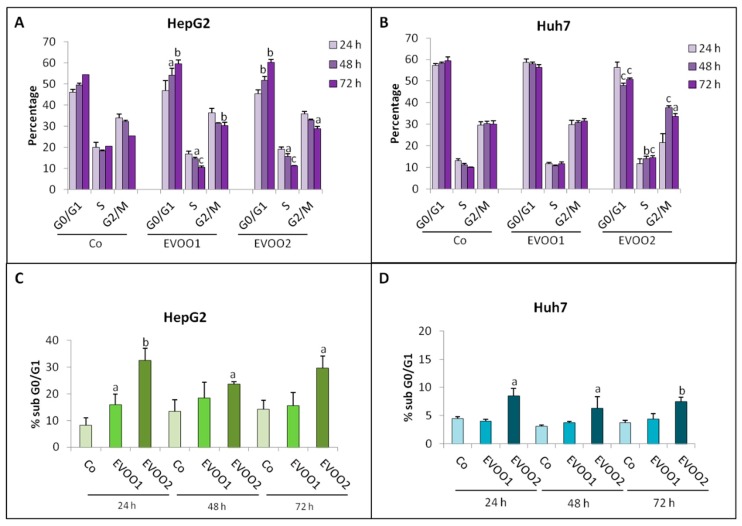
Phenolic extract alters cell cycle distribution of liver cancer cell. The cell lines were incubated for 24–72 h with two different doses of the EVOO extract. (**A**,**C**) Distribution in the cell cycle and percentage of cells in sub G0/G1 of HepG2 cell line. (**B**,**D**) Distribution in the cell cycle and percentage of cells in sub G0/G1 of Huh7 cell line. The results are representative of three experiments. Data are presented as mean ± SD. (a: *p* < 0.05, b: *p* < 0.01, c: *p* < 0.001 versus Control).

To understand if the reduced cell number reported in Figure 1A,B could result from the induction of apoptosis and/or from perturbations in the cell cycle progression, a flow cytometric analysis was performed. Independently from the extract dose, HepG2 cells showed a significant increase of the G0/G1 phase and a reduction of both S and G2/M phases (Figure 2A and Supplementary Figure S2A). In addition, the number of cells in sub G0/G1 region, where apoptotic cells normally are [18], significantly increased with respect to control values, with an effect that was persistent with EVOO2 (Figure 2C and Supplementary Figure S2C). As for Huh7 cells, a reduction in G0/G1 phase and an increase in S and G2/M phases were observed with EVOO2 only (Figure 2B and Supplementary Figure S2B). Similar to HepG2 cells, the Huh7 cultures also showed an increased number of cells in the sub G0/G1 region (Figure 2D and Supplementary Figure S2D). Similar observations were obtained on the Hep3B cell line (Supplementary Figure S1C–F).

**Figure 3:**
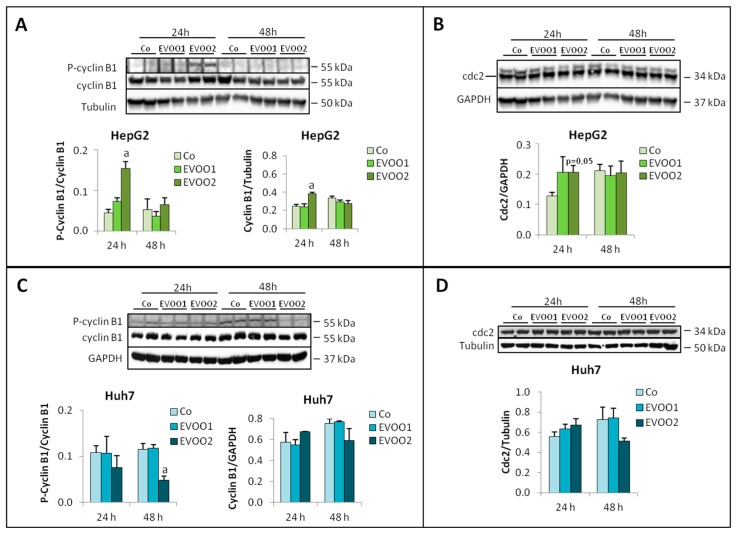
EVOO extract modulates the expression of the cyclinB1/cdc2 complex. The cell lines were incubated for 24 and 48 h with two different doses of the phenolic extract. (**A**,**B**) Expression of P-Cyclin B1 and cdc2 in HepG2 cell line. (**C**,**D**) Expression of P-Cyclin B1 and cdc2 in Huh7 cell line. The results are representative of three experiments. The protein molecular weights are reported. Data are presented as mean ± SD. (a: *p* < 0.05 versus Control).

The results reported above suggest that EVOO extract was able to affect both cell proliferation and death. However, despite the appearance of the sub G0/G1 peak, the amount of cleaved caspase-3, an accepted molecular marker of apoptosis [19], was not different between treated and untreated cells (Supplementary Figure S3). By contrast, significant modulations could be observed in the levels of molecules involved in regulating the cell cycle, in particular the cyclinB1/cdc2 complex that mediates the G2/M progression. In HepG2 cells, the expression of total and phosphorylated cyclin B1 significantly increased while cdc2 showed a trend to increase after 24 h treatment in the presence of EVOO2 (Figure 3A,B). Both proteins remained comparable to controls when exposed to EVOO1 (Figure 3A,B). No changes with respect to controls could be observed in Huh7 cultures, as for both cyclin B1 and cdc2 levels, with the exception of phoshorylated cyclin B1, that was significantly reduced after 48 h exposure to EVOO2 (Figure 3C,D).

**Figure 4:**
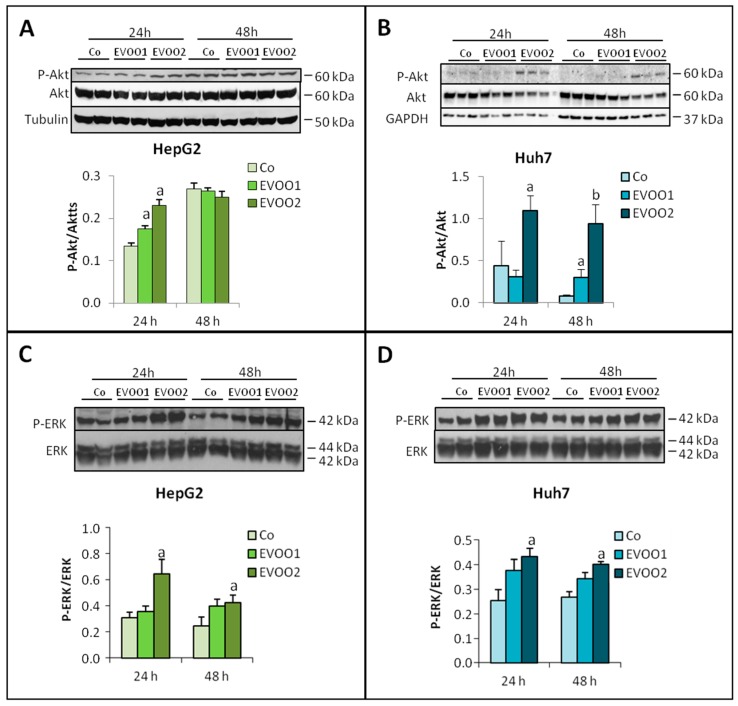
EVOO extract modulates the anabolic side of protein turnover. The cell lines were incubated for 24 and 48 h with two different doses of the phenolic extract. (**A**,**C**) Expression of P-Akt and P-ERK in HepG2 cell line. (**B**,**D**) Expression of P-Akt and P-ERK in Huh7 cell line. The results are representative of three experiments. The protein molecular weights are reported. Data are presented as mean ± SD. (a: *p* < 0.05, b: *p* < 0.01 versus Control).

Modulations of protein turnover could contribute to cell number reduction in cultures exposed to EVOO extract. Indeed, evidence has been provided suggesting that cancer growth can be modulated by the inhibition of intracellular proteolysis, mainly autophagy, and/or by stimulation of pathways involved in the enhancement of protein synthesis [20,21]. To clarify this point, the expression of molecules that are accepted markers of protein synthesis and degradation was evaluated in liver cancer cells treated with EVOO extract. Indirect insights into the modulations of protein synthesis can be obtained by analyzing one of the pathways, mainly regulating this aspect of intracellular protein metabolism, namely the Akt/mTOR one. In both HepG2 and Huh7 cells, the EVOO extract was able to significantly increase the amount of phosphorylated Akt (P-Akt) at 24 h. In HepG2 cultures, this effect was lost at 48 h (Figure 4A,B). Also, the levels of phosphorylated ERK (P-ERK) were significantly increased with respect to controls by EVOO2 in both cell lines (Figure 4C,D).

**Figure 5:**
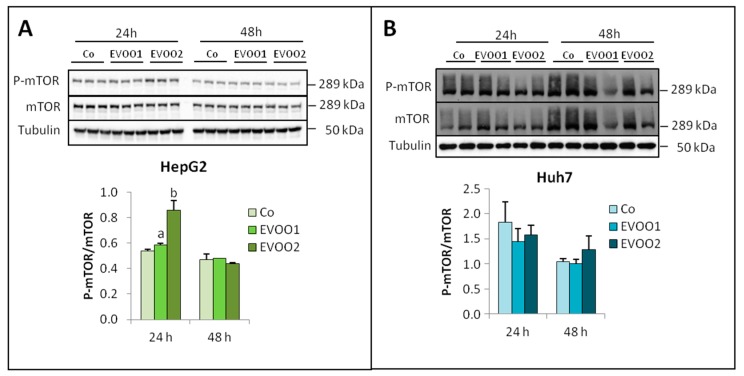
Phenolic extract modulates P-mTOR in HepG2 cell. (**A**) Expression of P-mTOR in HepG2 cell. (**B**) Expression of P-mTOR in Huh7 cell line. The cells were incubated for 24 and 48 h with two different doses of the EVOO extract. The results are representative of three experiments. The protein molecular weights are reported. Data are presented as mean ± SD. (a: *p* < 0.05, c: *p* < 0.001 versus Control).

Phosphorylated mTOR (P-mTOR) increased in HepG2 cells (Figure 5A) at both 24 h (EVOO2) and 48 h (EVOO1 and EVOO2), while in Huh7 cultures, no significant variations were observed (Figure 5B).

**Figure 6:**
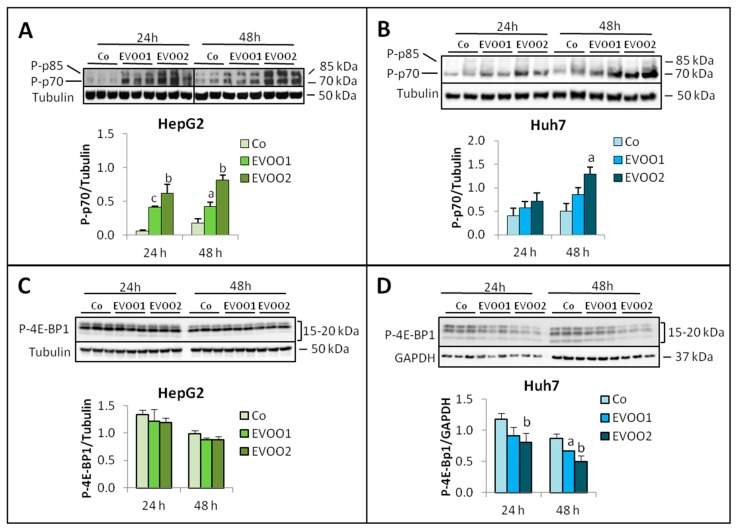
EVOO modulates the expression of P-p70 and P-4E-BP1. The cell lines were incubated for 24 and 48 h with two different doses of the phenolic extract. (**A**,**C**) Expression of P-p70 and P-4E-BP1 in HepG2 cell line. (**B**,**D**) Expression of P-p70 and P-4E-BP1 in Huh7 cell line. The results are representative of three experiments. The protein molecular weights are reported. Data are presented as mean ± SD. (a: *p* < 0.05, b: *p* < 0.01 versus Control).

Phosphorylated p70 (P-p70) increased in the presence of EVOO extract in both HepG2 (24 h and 48 h; Figure 6A) and Huh7 cells (48 h, EVOO2 only; Figure 6B). As for phosphorylated 4E-BP1 (P-4E-BP1), it was unchanged in HepG2 cells while it decreased significantly in Huh7 cultures at both 24 h (EVOO2) and 48 h (EVOO1 and EVOO2; Figure 6C,D).

**Figure 7:**
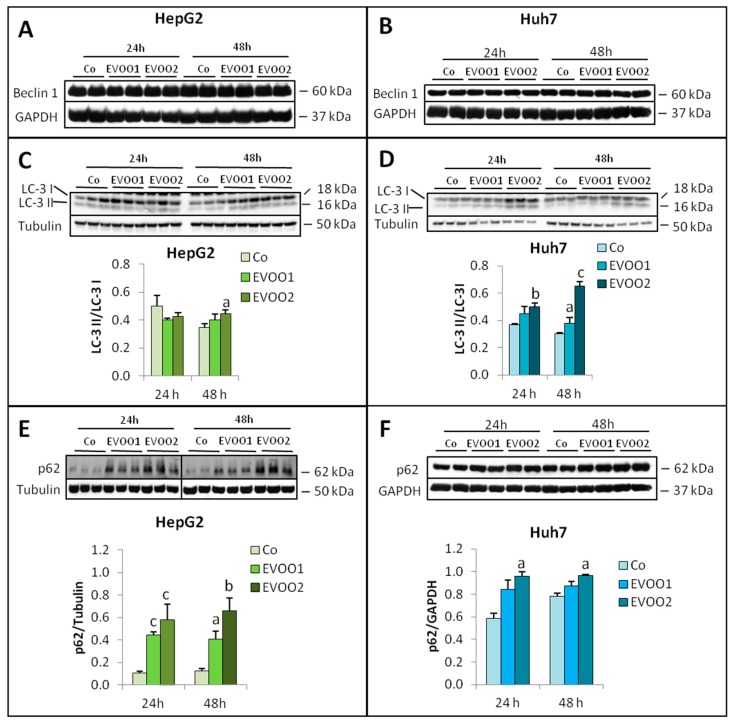
Phenolic extract modulates the expression of proteins involved in autophagy. The cells were incubated for 24 and 48 h with two different doses of the EVOO extract. (**A**,**C**,**E**) Expression of Beclin 1, LC-3 and p62 in HepG2 cell line. (**B**,**D**,**F**) Expression of Beclin 1, LC-3 and p62 in Huh7 cell line. The results are representative of three experiments. The protein molecular weights are reported. Data are presented as mean ± SD. (a: *p* < 0.05, b: *p* < 0.01, c: *p* < 0.001 versus Control).

Among the intracellular proteolytic systems, autophagy is the most relevant in terms of stress response. Indeed, this self-degradative lysosomal-mediated process is used by cells to remove misfolded or aggregated proteins, damaged organelles or intracellular pathogens and plays an important role in maintaining cellular homeostasis during stress [22,23]. Autophagy is persistently activated in rapidly growing tumors, allowing their survival in conditions of high metabolic demand and nutrient starvation. Since autophagy is responsive to stress conditions and has been shown to interfere with cancer growth [20], it was investigated in liver cancer cells exposed to EVOO extract by analyzing the expression of three accepted markers. In particular, beclin-1 and the conversion of LC-3 I to the lipidated moiety LC-3 II, both involved in autophagosome formation, and p62, that gives an estimate of autophagosome degradation, were assessed. Beclin-1 expression did not change in the presence of EVOO extract (Figure 7A,B), while a slight increase of the LC-3 II/LC-3 I ratio could be observed in both HepG2 and Huh7 cell lines exposed to EVOO2 (Figure 7C,D). As for p62, its levels increased in HepG2 cells at both time points, independently from the extract dose used (Figure 7E). Similar observations were obtained on the Hep3B cell line (Figure S4). In the Huh7 cell line, p62 expression increased at 24 and 48 h, only after exposure to EVOO2 (Figure 7F). These results suggest that autophagy is indeed activated above physiological levels in cultures exposed to EVOO, EVOO2 being on the whole more effective than EVOO1.

### 2.3. TNFα Potentiates the Cytotoxicity of EVOO Phenolic Extract

The results reported above show that the EVOO extract was able to obtain a partial inhibition of liver cancer cell growth. To investigate if this effect could be further enhanced, a combined approach coupling the EVOO extract with TNFα was tested. This latter is a pro-inflammatory cytokine known to exert cytotoxicity and previously demonstrated to potentiate the anti-tumor effect of taxol [17].

**Figure 8:**
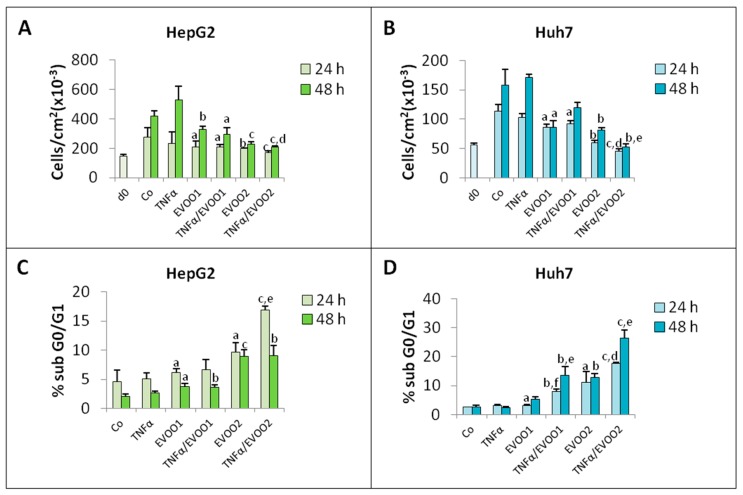
TNFα potentiates the cytotoxicity of EVOO phenolic extract. The cells were incubated for 24 and 48 h with two different doses of the phenolic extract in the presence or absence of TNFα. (**A**,**C**) Growth curve and percentage of sub G0/G1 of HepG2 cell line. (**B**,**D**) Growth curve and percentage of sub G0/G1 of Huh7 cell line. The results are representative of three experiments. Data are presented as mean ± SD. (a: *p* < 0.05, b: *p* < 0.01, c: *p* < 0.001 versus Control; d: *p* < 0.05, e: *p* < 0.01, f: *p* < 0.001 versus EVOO).

Consistent with previous observations [17,24], the presence of TNFα alone was not able to modify cell number as well as the number of cells in the sub G0/G1 region in both HepG2 and Huh7 cultures (Figure 8). By contrast, when the cytokine was added to cultures in the presence of EVOO, cell number was significantly reduced with respect to cultures exposed to the extract only (Figure 8A,B and Supplementary Figure S5A), while cells in the sub G0/G1 increased (Figure 8C,D). When combined with TNFα, the two EVOO extract doses appear to exert qualitatively comparable effects, in particular in Huh7 cultures (Figure 8). The addition of TNFα further supports the changes in cell cycle phase distribution induced by EVOO extract leading to an increased percentage of cells in G0/G1 and S phase (HepG2) and in G2/M (Huh7; Supplementary Table S1), confirming that the cytokine likely potentiates the antiproliferative effect of EVOO extract. Similar observations were also obtained on the Hep3B cell line (Supplementary Figure S5B).

**Figure 9:**
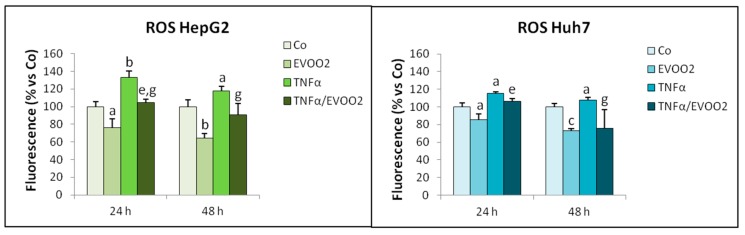
EVOO extract reduces basal ROS production. HepG2 and Huh7 cell lines incubated for 24 and 48 h with EVOO2 extract in the presence or absence of TNFα. The results are representative of three experiments. Data are presented as mean ± SD. (a: *p* < 0.05, b: *p* < 0.01, c: *p* < 0.001 versus Control; e: *p* < 0.01 versus EVOO; g: *p* < 0.05 versus TNFα).

Since the phenolic compounds present in EVOO are well known for their antioxidant properties, the extract used in this study was expected to prevent ROS production in the presence of an oxidative stimulus as TNFα is. Indeed, the increased ROS release induced by TNFα alone in both HepG2 and Huh7 cultures was significantly reduced by the exposure to EVOO2, which was also able to reduce basal ROS production (Figure 9).

**Figure 10:**
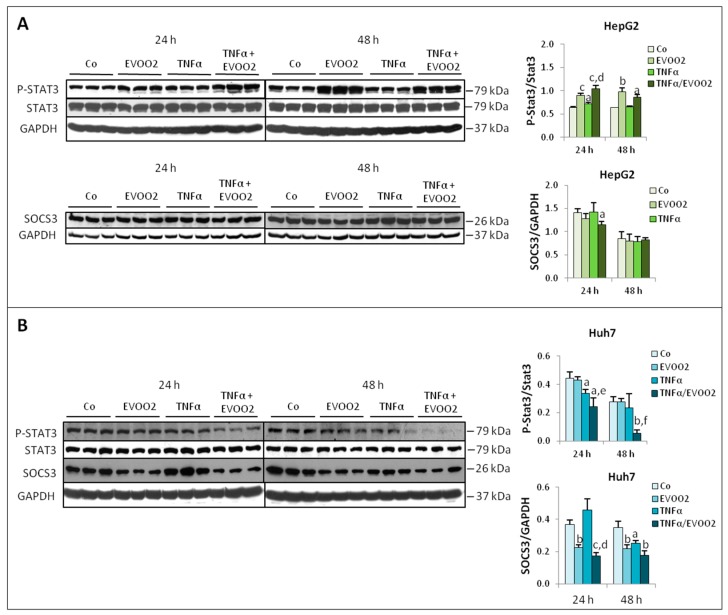
The combined treatment of EVOO and TNFα modulates the expression of P-STAT3 and SOCS3. The cell lines were incubated for 24 and 48 h with EVOO2 extract in the presence or absence of TNFα. (**A**) Western blot analysis of P-STAT3 and SOCS3 in HepG2 cell. (**B**) Expression of P-STAT3 and SOCS3 in Huh7 cell line. The results are representative of three experiments. The protein molecular weights are reported. Data are presented as mean ± SD. (a: *p* < 0.05, b: *p* < 0.01, c: *p* < 0.001 versus Control; d: *p* < 0.05, e: *p* < 0.01, f: *p* < 0.001 versus EVOO).

Previous observations showed that the enhancement of cytotoxicity induced in HCC cell lines by the combined action of TNFα and taxol was associated with inhibition of STAT3 activation and with reduced SOCS3 expression, this latter playing a causative role [17]. To assess if the same mechanism could also be operating in the cytotoxicity induced by the EVOO extract combined with TNFα, STAT3 phosphorylation and SOCS3 expression were assessed. Exposure of HepG2 cells to the combined treatment with EVOO2 extract and TNFα for 24 h was able to further increase the overexpression of phosphorylated STAT3 induced by the extract alone, while it reduced the levels of SOCS3 with respect to controls (Figure 10A). Such effects were lost at 48 h, where SOCS3 remained comparable to controls while P-STAT3 expression was higher than controls in EVOO2-treated cultures, but unmodified by the presence of TNFα (Figure 10A). As for Huh7 cells, the combined treatment resulted in progressively reduced P-STAT3 and SOCS3 levels. These latter were also decreased by the extract alone at both 24 and 48 h, or by TNFα alone at 48 h culture (Figure 10B).

**Figure 11:**
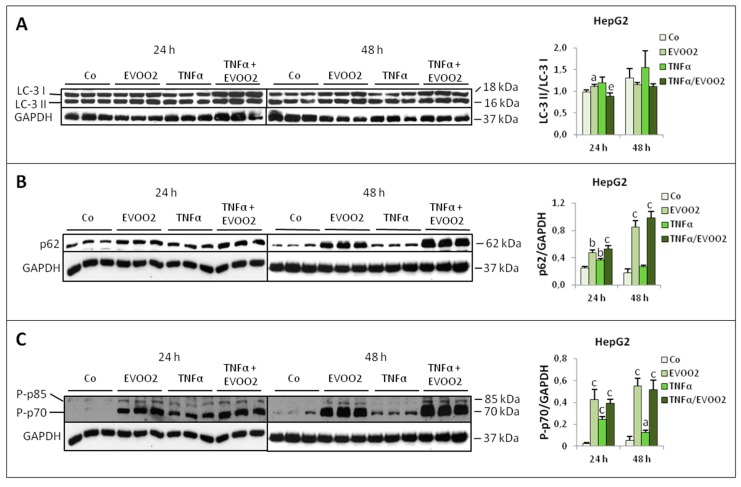
The combined treatment of EVOO extract and TNFα modulates protein turnover in HepG2 cells. (**A**–**C**) Expression of LC-3 (**A**), p62 (**B**) and P-p70 (**C**) in HepG2 cell lines incubated for 24 and 48 h with EVOO2 extract in the presence or absence of TNFα. The results are representative of three experiments. The protein molecular weights are reported. Data are presented as mean ± SD. (a: *p* < 0.05, b: *p* < 0.01, c: *p* < 0.001 versus Control; e: *p* < 0.01 versus EVOO).

Finally, in the HepG2 cells the combined treatment with EVOO2 and TNFα reduced the LC-3 II/LC-3 I ratio below control and extract alone levels (Figure 11A), while did not affect the increased p62 and P-p70 expression induced by EVOO2 or TNFα (Figure 11B,C).

**Figure 12:**
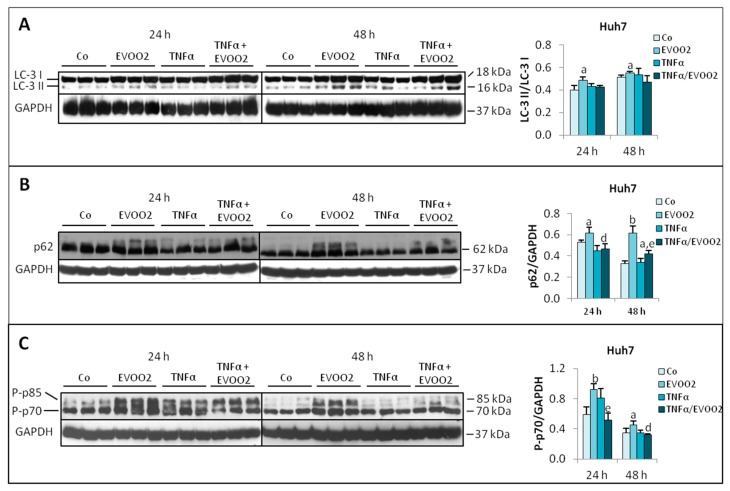
The combined treatment of EVOO extract and TNFα modulates protein turnover in Huh7 cells. (**A–C**) Expression of LC-3 (**A**), p62 (**B**) and P-p70 (**C**) in Huh7 cell lines incubated for 24 and 48 h with EVOO2 phenolic extract in the presence or absence of TNFα. The results are representative of three experiments. The protein molecular weights are reported. Data are presented as mean ± SD. (a: *p* < 0.05, b: *p* < 0.01 versus Control; d: *p* < 0.05, e: *p* < 0.01 versus EVOO).

As for Huh7 cells, the LC-3 II/LC-3 I ratio was unchanged with respect to control values in the presence of the combined treatment, while it increased when EVOO2 only was added to the culture medium (Figure 12A). The extract alone markedly increased the levels of p62, which were significantly reduced by the association of EVOO2 with TNFα (Figure 12B). Finally, the combined treatment with EVOO2 and TNFα was able to prevent the increased expression of P-p70 induced by the extract alone (Figure 12C). Similar observations were also obtained on the Hep3B cell line (Supplementary Figure S5C).

## 3. Discussion

The present study shows that exposure of liver cancer cell lines to an EVOO extract particularly enriched in LA (67%) and OC (32%) results in inhibition of cell proliferation that is associated with induction of autophagy and that can be potentiated by the pro-inflammatory cytokine TNFα.

Several studies have already shown that the growth of breast, prostate, pancreatic and many other cancer cell lines can be impaired by OC [25,26,27]. In this scenario, however, very few data are available about the effects exerted by OC on liver cancer cell lines. Just a couple of rather recent reports are available. One is showing that OC exerts antitumor effects on a panel of human HCC cell lines by inducing ROS production [10]. According to the other report, reduced proliferation and increased apoptosis occur in HCC cell cultures exposed to OC. The same authors also show that OC inhibits tumor growth in an orthotopic HCC model [11].

The results reported in the present study conform to previous data, showing that the growth of both HepG2 and Huh7 cell lines is impaired in the presence of EVOO extract. Such impairment is associated with an altered cell cycle distribution that is peculiar to the specific cell line. The accumulation of Huh7 cells in the G2/M phase is consistent with reduced levels of phosphorylated cyclin B1, that is required for cyclin B1/cdc2 complex translocation to the nucleus and the consequent transition from G2 to M phase [28]. The exposure of HepG2 cells to EVOO extract results in the accumulation in the G0/G1 phase of the cell cycle, consistently with observations reported by Pei et al. [11]. A similar pattern was reported in human breast cancer cells, where OC exposure resulted in G1/M arrest that was associated with modulations of cyclin D1, p21 and p27 expression [29]. In the present study, 24 h EVOO2 treatment of HepG2 cells results in increased levels of the ratio between phosphorylated and total cyclin B1, associated with a trend towards increased cdc2 expression. Such a pattern could be viewed as a sort of compensatory mechanism trying to force the progression into the cell cycle. The altered cell cycle distribution caused by EVOO extract in liver cancer cell lines is paralleled by an increased number of cells in the sub G0/G1 region, e.g., cells with DNA content < 2n. The appearance of these cells could derive from EVOO extract-induced DNA damage and subsequent degradation. In this regard, several studies have excluded the possibility that EVOO-derived compounds may exert a genotoxic action [reviewed in 13]. However, another study proposed that very high concentrations of different EVOO extracts could exert a damaging action on DNA, enhancing cytotoxicity [30]. Another possibility is that the increased percentage of cells in the sub G0/G1 region reflects death by apoptosis. Different from the results reported by [10,11], this is not associated with caspase 3 activity above control levels. Several reasons could explain this discrepancy: (a) the EVOO extract used in the present study contains LA in addition to OC, (b) the nominal OC concentrations used in the present study are 5 and 10 μM, while the results reported by [10,11] were obtained with pure OC, used at least at 25 μM concentration. In this regard, experiments performed with commercially available OC show that cytotoxicity in both HepG2 and Huh7 cells is not evident unless concentrations of 100 μM and 50 μM are used, respectively (Supplementary Figure S6), (c) apoptosis can occur also in the absence of caspase 3 activation [31,32,33].

The modulations of cell proliferation and death reported above could depend on pathways involved in the regulation of protein metabolism. In this regard, the present study focused on: (a) the Akt/mTOR pathway, mainly involved in the regulation of anabolic signaling, including protein synthesis; (b) autophagy, the intracellular proteolytic system mainly in charge of facing stress conditions. These pathways are strictly interconnected: activation of the Akt/mTOR signaling is generally associated with down-regulation of autophagy [20,21]. According to the results shown in the present study, the Akt-mTOR-p70 pathway is not down-regulated in HepG2 and Huh7 cells exposed to EVOO extract. This result is in contrast with those reported on malignant human melanoma and multiple myeloma cells, where OC was shown to inhibit cell proliferation and to induce apoptosis by reducing the AKT/ERK-dependent signaling pathways [12,34]. On the other side, the levels of P-4E-BP1 are significantly decreased in the Huh7 cells treated with EVOO extract, suggesting that protein synthesis might indeed be inhibited and that other pathways in addition to the one regulated by mTOR-p70 likely contribute to regulate the amount of P-4E-BP1 [35].

Besides protein synthesis, degradation rate is also relevant to protein metabolism. In this regard, interference with the intracellular proteolytic systems is known to contribute to cell death [36]. The activation of autophagy above physiological levels, in particular, was shown to promote or suppress tumor development, providing a sort of ‘double-edged sword’, depending on tumor type and stage. The present study reports for the first time that the LC-3 II/LC-3 I ratio, an accepted marker of autophagic sequestration, is increased in liver cancer cell lines exposed to EVOO extract. In both HepG2 and Huh7 cultures, the altered LC-3 II/LC-3 I ratio is associated with high levels of p62, suggesting that unprocessed autophagosomes are likely accumulating, possibly in view of exhaustion of the lysosomal clearance capacity. Another possibility is that increased p62 levels could reflect transcriptional induction, as previously reported [37]. The activation of autophagy in HCC cell lines was proposed to play a role in chemoresistance, a frequent occurrence in HCC that largely compromises patient outcome. As an example, autophagy induced by tumor-associated macrophages protects Huh7 cells against oxaliplatin-dependent cytotoxicity. Such protection is abrogated when autophagy is inhibited by ATG5 silencing [38]. Along this line, the present results suggest that liver cancer cells try to face the challenge imposed by EVOO extract by activating autophagy above control levels (increased LC-3 II/LC-3 I ratio) but are unable to reach complete autophagosome degradation (high p62 levels) and eventually die.

The contribution of components of the inflammatory response such as macrophages and cytokines in modulating chemoresistance is widely accepted, and several reports show that cytotoxicity of anti-cancer drugs can be enhanced in the presence of pro-inflammatory cytokines [39,40]. As an example, taxol-induced cell death in both HepG2 and Huh7 cultures is higher in the presence than in the absence of TNFα [17]. The results reported in the present study show for the first time that also the cytotoxic effect exerted in liver cancer cell lines by EVOO extract can be potentiated by TNFα, otherwise ineffective. Potential mechanisms of this enhanced effect could be, among others, the induction of oxidative stress, the modulation of STAT3-dependent signaling and the interference with autophagic degradation.

Secoiridoids contained into olive oil, including OC and LA, were shown to directly protect cells from oxidative stress by working as free radical scavengers as well as by upregulating the Nrf2-dependent antioxidant signaling pathway [14,41]. On the other side, they appeared to inhibit cancer cell proliferation and to induce cell death by increasing ROS production and/or by hampering the intracellular antioxidant defense. In addition, cell death could also be promoted by inducing mitochondrial and lysosomal dysfunction [14]. The EVOO extract used in the present study is able to decrease ROS release in both HepG2 and Huh7 cells, a pattern that is even more evident when cultures are exposed to the pro-inflammatory cytokine TNFα, a well-known pro-oxidant stimulus. These data are in contrast with those reported by Cusimano et al. [10], who showed that OC-induced cell growth arrest in HCC cell lines was associated with enhanced ROS production. As discussed above, the experimental conditions used in the present study are quite different from those adopted by [10]. The data here reported clearly rule out the possibility that the increased cytotoxicity occurring in liver cancer cell lines exposed to the combined action of EVOO extract and TNFα might derive from enhanced oxidative stress.

Recently the anticancer effect of OC on HCC cells was ascribed to its ability to inhibit the transcription factor STAT3 [11], that contributes to several processes such as cell proliferation, differentiation, survival, and death. STAT3 can be activated by cytokines but was also shown to be constitutively phosphorylated in many tumors, also depending on mTOR activity [42]. Along this line, STAT3 inhibition frequently resulted in impaired tumor growth [43]. Consistently, the potentiation of cell death induced in liver cancer cell cultures by the combined treatment with taxol and TNFα was associated with reduced levels of both phosphorylated STAT3 and SOCS3, one of the STAT3 target genes [17]. The results reported in the present study, however, only partially conform to these findings. Indeed, a reduction of P-STAT3 and SOCS3 levels can be observed in Huh7 cells in the presence of EVOO extract + TNFα, consistently with the enhanced cell death. By contrast, despite the occurrence of cytotoxicity, P-STAT3 expression is higher in HepG2 cultures exposed to EVOO than in controls, irrespective of the presence of TNFα, and only slight changes can be observed for SOCS3. The discrepancy between HepG2 and Huh7 cell lines could be due to different reasons: (a) mTOR activation (phosphorylation) is higher than controls in HepG2 cells exposed to EVOO extract, but not in the Huh7 cultures, and (b) the degree of differentiation of the two cell lines (HepG2 more differentiated than Huh7) [44,45,46] might account for the different response to EVOO extract and TNFα, at least in terms of STAT3-dependent signaling.

As reported above, the inability to complete autophagic degradation could be one of the mechanisms leading to cell death in the presence of EVOO extract. The increased cytotoxicity induced by the addition of TNFα could result from further modulations of autophagy. Indeed, the combined treatment effectively reduces the increased LC-3 II/LC-3 I ratio induced by EVOO2. In the absence of information about the autophagic flux, that is one of the main limitations of the present study, these observations suggest that the treatment of liver cancer cells with EVOO extract + TNFα leads to reduced autophagic sequestration, likely exposing cells to the detrimental action of unwanted substrates such as damaged proteins and organelles. In this regard, the cytokine is worsening the picture induced by EVOO extract: while the latter blocks the completion of autophagic degradation, addition TNFα also impinges on substrate sequestration and autophagosome formation.

## 4. Materials and Methods

### 4.1. Chemicals and Antibodies

All reagents, unless otherwise indicated, were purchased by Sigma-Aldrich, Italy. The acetonitrile and n-hexane used for the extraction and purification of olive oil were from Carlo Erba Reagents (Milan, Italy). The primary antibodies against caspase-3, phospho-p70 (Thr421/Ser424), phospho-4E-BP1 (Thr37/46), phospho-STAT3 (Tyr705), STAT3, SOCS3, phospho-ERK (Thr202/Tyr204), ERK, phospho-AKT (Ser473), AKT, phospho-mTOR (Ser2448) were purchased by Cell-Signaling Technology, Danvers, MA, USA. The primary antibodies against mTOR, cyclin B1 and cdc2 were purchased by SantaCruz Biotechnology, Dallas, TX, USA. The primary antibodies against LC-3, GAPDH and tubulin were purchased by Sigma-Aldrich, Milan, Italy.

### 4.2. Phenolic Extract Preparation

The EVOO used for the extraction was freshly produced in September 2015 from olives (*Olea europaea* L.) harvested in Racalmuto (Lat. 37.4076652 Long. 13.7330745; Sicily, Italy). For the extraction and purification of phenolic substances, and in particular of the oleocanthal, was used the protocol reported by Montedoro et al. [47], with some modifications. In particular, a liquid/liquid extraction was carried out using 300 g of extra virgin olive oil and 300 mL of acetonitrile in a separating funnel. The mixture was homogenized by shaking vigorously for 2 min and left to rest until the 2 phases were completely separated. The organic phase was withdrawn and 300 mL of acetonitrile were added again in order to repeat the extraction as described above (final extraction ratio 1:2 w:v). The acetonitrile extracts were collected in a single fraction and purified by low-pressure column chromatography (1.8 cm Ø × 48 cm h) using acetonitrile as eluent and a stationary phase consisting of a polar amorphous resin (silica gel 60; 0.040–0.063 mm, Merck, Branchburg, NJ, USA). The eluate was collected in one purified fraction that was vacuum concentrated up to 5 mL by rotary evaporator (Rotavapor^®^; Buchi, Flawil, Switzerland) at 30 °C. The concentrated extract was further purified to remove traces of lipids by washing with *n*-hexane in a 1:1 v:v ratio. The sample was vortexed for 2 min and centrifuged for 5 min at 5000× *g* to separate the *n*-hexane. This washing procedure was repeated 3 times. The extract was centrifuged at 10,000× *g* for 2 min at room temperature to remove the residual impurities. The sample was then completely dried under nitrogen flow at room temperature. The extract obtained was resuspended in 9 mL of DMSO and used for quantification and biological assays.

### 4.3. High Performance Liquid Chromatography (HPCL) Analysis

To identify the phenolic compounds, a portion of the extract in DMSO was diluted 1:2 with acetonitrile. Qualitative and quantitative analyses were performed by HPLC (1200-HPLC, Agilent Technologies, Santa Clara, CA, USA) coupled with diode-array detector (DAD) (Agilent Technologies, Santa Clara, CA, USA) and 6330 Series Ion Trap Mass Spectrometer (Agilent Technologies, Santa Clara, CA, USA). The HPLC was equipped with a Kinetex reverse phase C18 column (2.6 μM, 100 × 3.0 mm) (Phenomenex, Torrance, CA, USA). The chromatographic column was conditioned with the following solvent mixture: Solvent A—ultra pure MilliQ water (Millipore, Temecula, CA, USA) and formic acid 0.1% (*v*/*v*), Solvent B—acetonitrile with formic acid 0.1% (*v*/*v*). The initial solvent composition was 80% solvent A and 20% solvent B at constant flow of 0.2 mL/min.

For the chromatographic separation the following solvent gradient was used: at 40 min solvent B was 60%, from 41 min to 54 min, solvent B reached the 98%, at 46 min, solvent B was 20%. The chromatographic column was reconditioned before the next analysis for 10 min. The eluted compounds were monitored by DAD at a wavelength of 278 nm and identified by mass spectrometry with a detector equipped with Electrospray Ionization Source (ESI) operating in negative mode. Qualitative analyses were performed in tandem mass spectrometry (MS/MS) (50–600 *m*/*z*). Quantitative analyses were conducted in Multi Reaction Monitoring (MRM) by selecting the following molecular ions: [M − H]^−^ 303 *m*/*z* (for oleocanthal), [M − H]^−^ 361 m/z (ligstroside aglycone), [M − H]^−^ 377 *m*/*z* (oleuropein aglycone), [M − H]^−^ 241 *m*/*z* (elenolic acid), [M − H]^−^ 353 m/z (chlorogenic acid, external standard). The quantification was carried out through an external calibration curve with chlorogenic acid. The identification was possible by comparison of fragmentation profile with literature data [48].

### 4.4. Cell Culture and Treatments

Huh7 and Hep3B cells, a well differentiated HCC lines, are characterized by an epithelial-like morphology (JCRB Cell Bank, Osaka, Japan and ATCC, Rockville, MD, USA, respectively). Hep3B, HepG2 cells, isolated from a hepatoblastoma (ATCC, Rockville, MD, USA), retaining features of differentiated hepatocytes and an epithelial morphology. The three different human liver cancer cell lines (HepG2, Huh7 and Hep3B) were grown at 37 °C in the presence of CO_2_ 5% (incubator HERAcell 150i, Thermo Scientific, Waltman, MS, USA) in Dulbecco’s Modified Eagle’s Medium (DMEM) supplemented with 10% fetal bovine serum, 1% penicillin and streptomycin (PES). Liver cancer cells were treated with different concentrations of phenolic extract at 24, 48 and 72 h (growth curve, morphology, cell cycle analysis) or at 24 or 48 h (western blotting, ROS determination). The EVOO concentrations indicated in the experiments refer to the amount of OC present in the phenol extract (5 uM in EVOO1 and 10 uM in EVOO2) and were chosen on the basis of previously published data [11,25,26,27] and of a pilot dose-response experiments (Supplementary Figure S7). Since the phenolic extract was resuspended in DMSO, the same amount of DMSO was also added to control cells and to those treated with 5 μM concentration. The DMSO did not exceed the final 0.7%. During the experiments, the culture medium containing DMSO or the phenolic extract was substituted daily. Cells were not synchronized before exposure to the EVOO extract. Cells were counted by a hemocytometer Burker chamber. Cell suspensions have been appropriately diluted in order to obtain no more than 30–40 cells/field. Such dilution was then taken into account when calculating cell concentration. Trypan Blue dye was used to assess cell viability. Cell samples were diluted by preparing a 1:1 dilution of the cell suspension using a 0.4% Trypan Blue solution. Non-viable cells will be blue, viable cells will be unstained. Only a few cells (2–3 cells per counting field) were positive for Trypan Blue.

### 4.5. Morphological Analysis

Cell cultures were observed using a reversed phase-contrast optical microscope Eclipse TS100 (Nikon Instruments, Amsterdam, The Netherlands) and photographed (digital microscope camera, Ec3, Leica Microsystems, Wetzlar, Germany).

### 4.6. Cell Cycle Analysis

Cell cycle progression and DNA distribution were analyzed by flow cytometry using propidium iodide staining. Cells were washed with PBS-EDTA, trypsinized and centrifuged (500× *g*, 10 min at 4 °C in ALC centrifuge model 4227R) to collect living cells. To recover the apoptotic bodies, the culture medium was centrifuged at 1500× *g* at 4 °C for 10 min. After washing, cells and apoptotic bodies were separately fixed in ice cold 70% *v*/*v* ethanol for at least 30 min at Room Temperature then centrifuged as above, put together in PBS containing DNase-free RNase A (0.4 g/mL) and incubated for 30 min. Finally, cells were incubated 10 min with propidium iodide (0.18 mg/mL) and analyzed by flow cytometry using the BD Accuri™ C6 (Becton Dickinson, Franklin Lakes, NJ, USA). The apoptotic population was estimated by evaluating the percentage of cells characterized by a < 2n DNA fluorescence (hypodiploid or sub G0/G1 cells) [18] and analyzed using the FCS Express 4 (De Novo Software, Pasadena, CA, USA).

### 4.7. ROS Determination

The intracellular accumulation of ROS was determined using the fluorescent probe 2′,7′-difluorodihydrofluorescein diacetate (DCFH-DA; Molecular Probes, OR, USA). This probe is transported across the cell membrane and cleaved by non-specific esterases to form DCFH, which is further oxidized by ROS to form the fluorescent compound (DCF). DCFH is able to react with different radical species, such as organic hydroperoxides and in particular with lipid hydroperoxides [49]. Cells were treated as indicated and then incubated with DCFH-DA 3.5 µM for 30 min at 37 °C in the dark. After incubation, cells were washed, and the level of DCF fluorescence was determined using the Becton Dickinson Accuri™ C6 (Becton Dickinson, Franklin Lakes, NJ, USA). The intensity of the fluorescence is proportional to the amount of ROS present into the sample.

### 4.8. Western Blotting

Cells were lysed in RIPA buffer (1% NP40, 0.5% NaDOC, 0.1% SDS) supplemented with 1 mM DTT, 0.1 mM PMSF, 2 mg/mL aprotinin, 2 mg/mL leupeptin, 100 mM NaF. Protein content was determined using Bio-Rad Protein Assay kit (Bio-Rad, Franklin Lakes, CA, USA). Equal amounts of protein (30 μg) were fractionated on polyacrilamide SDS-gel and transferred to nitrocellulose membrane (BioRad, Franklin Lakes, CA, USA). The membranes were blocked with a solution containing 5% non-fat milk (or 5% BSA for phosphorylated protein) in TBS 0.05%-Tween and incubated with primary antibodies overnight at 4 °C. Subsequently, the membranes were incubated with secondary antibody coupled with horseradish peroxidase (Bio-Rad, Franklin Lakes, CA, USA). Reactive proteins were visualized using ECL Western Blotting Detection kit (Amersham Bioscences, Buckinghamshire, UK). The filter was placed in contact with a chemiluminescent plate (Hyperfilm ECL, Amersham Bioscences, Buckinghamshire, UK). Depending on the target protein, GAPDH or tubulin expression were used as loading control. Densitometry was assessed using the TotalLab software (NonLinear Dynamics, Quayside, UK). 

### 4.9. Statistical Analysis

All values are expressed as the mean ± standard deviation (SD). The significance among mean values was evaluated using the two-tailed unpaired Student’s *t*-Test (*p* < 0.05).

## 5. Conclusions

In conclusion, the results reported in the present study show that treatment of liver cancer cells with an EVOO extract enriched in OC and LA reduces cell proliferation and increases cell death. This is the first time that the action of LA is also taken into consideration when studying the effectiveness of EVOO derived polyphenols. The results here reported suggest that the presence of LA lowers the concentration of OC required for cytotoxicity, at least in liver cancer cell lines. In addition, these results for the first time show that the effects of EVOO extract can be potentiated by the pro-inflammatory cytokine TNFα and that modulations of autophagy likely account for these effects.

## Figures and Tables

**Table 1:** Fragmentation profile of identified olive oil polyphenols. (**A**) Reverse phase HPLC–DAD-ESI-MS/MS, (**B**) Percentage quantification.

**(A)**		
**Compounds**	**MS (M − H)**	**MS/MS (M − H)**
Oleocanthal	303 *m*/*z*	
		290.9 *m*/*z*
		240.8 *m*/*z*
		258.9 *m*/*z*
Ligstroside aglycone	361 *m*/*z*	
		290.9 *m*/*z*
		240.8 *m*/*z*
		258.9 *m*/*z*
Elenolic acid	241 *m*/*z*	
		138.8 *m*/*z*
		194.8 *m*/*z*
		126.9 *m*/*z*
		169.7 *m*/*z*
		222.8 *m*/*z*
		100.9 *m*/*z*
Oleuropein aglycone	377 *m*/*z*	
		306.9 *m*/*z*
		274.9 *m*/*z*
		256.8 *m*/*z*
		194.8 *m*/*z*
		345.0 *m*/*z*
**(B)**		
**B. Quantitative results**		**(% p/p)**
Oleocanthal		31.8
Ligstroside aglycone		67.0
Elenolic acid		0.6
Oleuropein aglycone		0.6

## Supplementary Materials

The following are available online at https://www.mdpi.com/2072-6694/11/11/1640/s1, Figure S1: Growth curve, morphological analysis and cell cycle distribution of Hep3B cell line incubated for 24-72 h with two doses of EVOO, Figure S2: Cytograms of HepG2 and Huh7 cell lines incubated for 24-72 h with different doses of EVOO, Figure S3: Expression of caspase-3 in HepG2 (A) and Huh7 (B) cell lines incubated for 24 and 48 h with two different doses of the phenolic extract, Figure S4: Expression of p62 in Hep3B cell line incubated for 24 and 48 h with two doses of EVOO, Table S1: Flow cytometric analysis of HepG2, Huh7 and Hep3B cell lines incubated for 24 and 48 h with different doses of the phenolic extract in the presence or absence of TNFα, Figure S5: Growth curve, percentage of subG0/G1 and expression of P-p70 in Hep3B cell lines incubated for 24 and 48 h with phenolic extract in the presence or absence of TNFα, Figure S6: Growth curve of HepG2 and Huh7 cell lines incubated for 48 h with three different doses of oleocanthal, Figure S7: Dose-response experiments of HepG2 and Huh7 cell lines incubated for 24–72 h with different doses of EVOO.

## Abbreviations

TNFα	tumor necrosis factor α
Akt	protein kinase B
mTOR	mammalian target of rapamycin
ERK	extracellular signal-regulated kinases
4E-BP1	eukaryotic translation initiation factor 4E (eIF4E)-binding protein 1
ROS	reactive oxygen species
SOCS3	suppressor of cytokine signaling 3
LC-3	microtubule-associated protein 1A/1B-light chain 3
GAPDH	glyceraldehyde 3-phosphate dehydrogenase
SDS	sodium dodecyl sulfate
