# Supplementary material for: Anti-Proliferative Effects of an Extra-Virgin Olive Oil Extract Enriched in Ligstroside Aglycone and Oleocanthal on Human Liver Cancer Cell Lines
Source: Cancers (Basel). 2019 Oct 24;11(11):1640. doi: 10.3390/cancers11111640 (PMC6896128; doi:10.3390/cancers11111640)
Supplement: Supplementary file 1 [file cancers-11-01640-s001.zip › Figure S1.pdf]

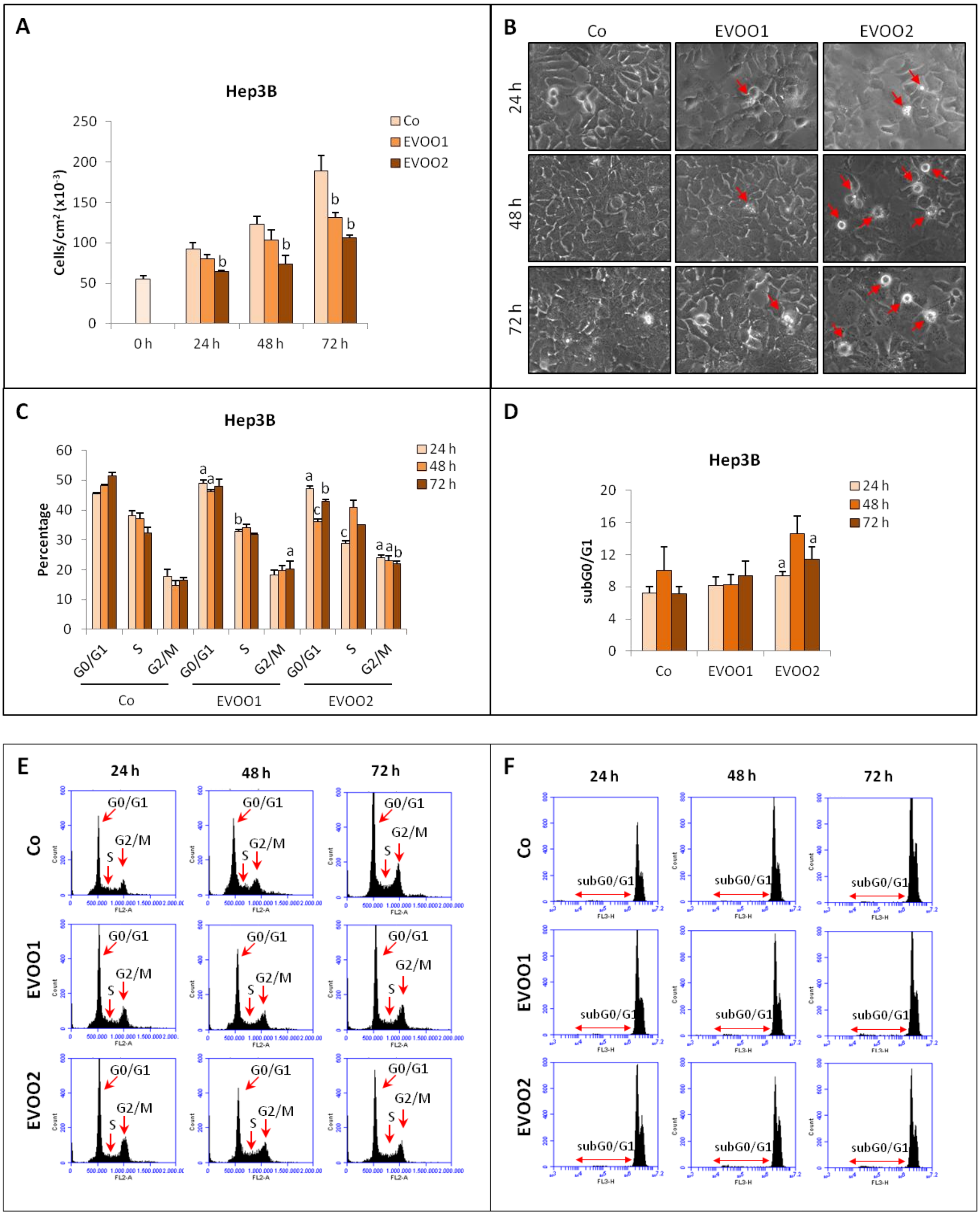

Figure S1: (A) Growth curve, (B) morphological analysis, (C) cell cycle distribution, (D) percentage of cells in sub G0/G1, (E, F) cytograms of cell cycle and subG0/G1 of Hep3B cell line incubated for 24-72 h with two doses of EVOO. The results are representative of three experiments. Data are presented as mean ± SD. (a p<0,05, b p<0,01, c p<0,001 vs Co).
