# Supplementary material for: Anti-Proliferative Effects of an Extra-Virgin Olive Oil Extract Enriched in Ligstroside Aglycone and Oleocanthal on Human Liver Cancer Cell Lines
Source: Cancers (Basel). 2019 Oct 24;11(11):1640. doi: 10.3390/cancers11111640 (PMC6896128; doi:10.3390/cancers11111640)
Supplement: Supplementary file 1 [file cancers-11-01640-s001.zip › Figure S3.pdf]

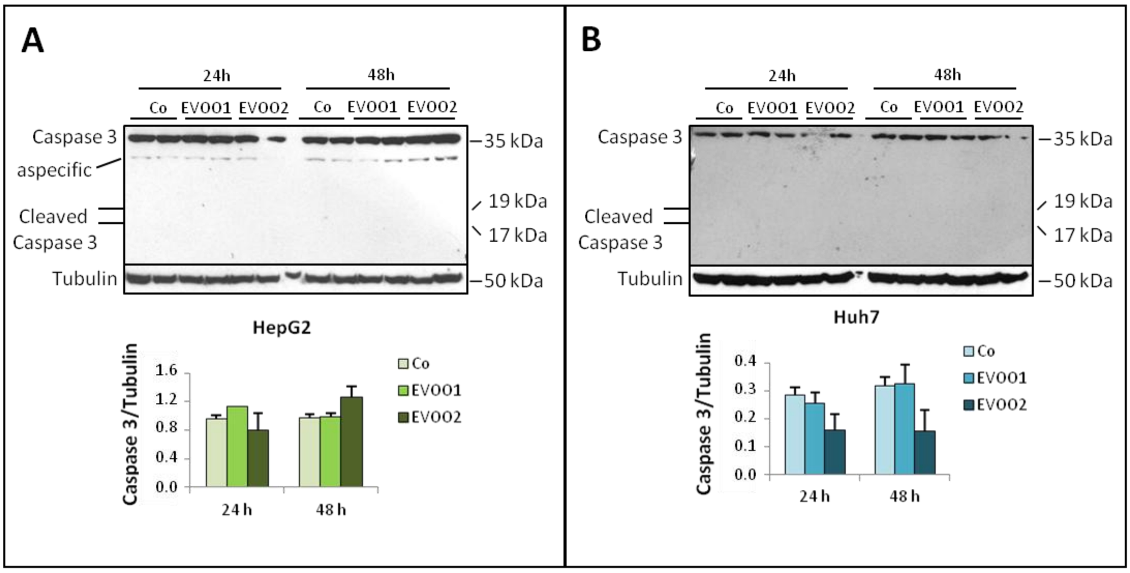

Figure S3: Expression of caspase-3 in HepG2 (A) and Huh7 (B) cell lines incubated for 24 and 48 h with two different doses of the phenolic extract. The protein molecular weights are reported.
