# Supplementary material for: Anti-Proliferative Effects of an Extra-Virgin Olive Oil Extract Enriched in Ligstroside Aglycone and Oleocanthal on Human Liver Cancer Cell Lines
Source: Cancers (Basel). 2019 Oct 24;11(11):1640. doi: 10.3390/cancers11111640 (PMC6896128; doi:10.3390/cancers11111640)
Supplement: Supplementary file 1 [file cancers-11-01640-s001.zip › Figure S5.pdf]

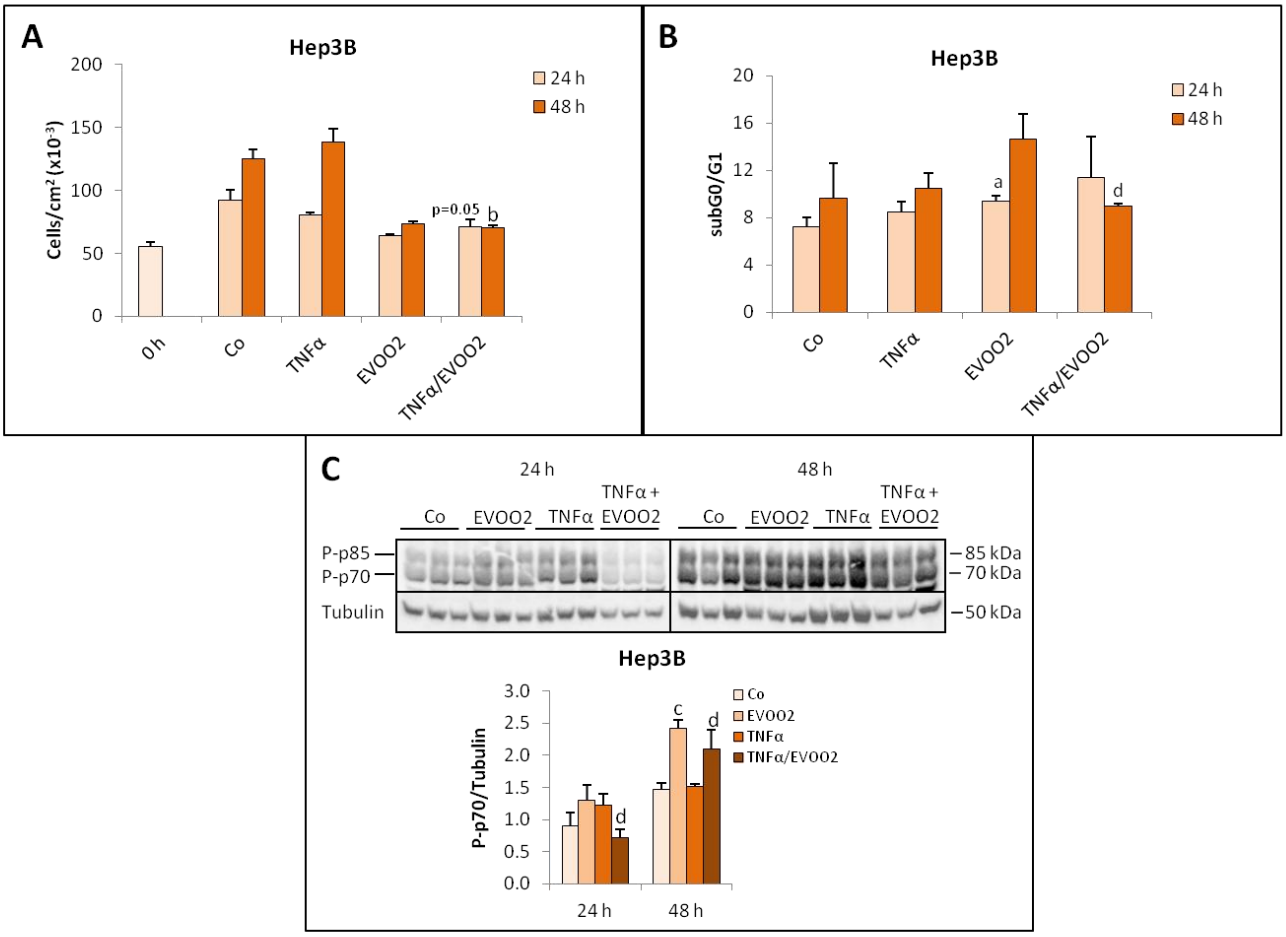

Figure S5: (A) Growth curve, (B) percentage of subG0/G1 and (C) expression of P-p70 in Hep3B cell lines incubated for 24 and 48 h with phenolic extract in the presence or absence of TNFα. The results are representative of three experiments. The protein molecular weights are reported. Data are presented as mean ± SD. (a $p<0,05$, b $p<0,01$, c $p<0,001$ vs Co; d $p<0,05$ vs EVOO).
