# Supplementary material for: Anti-Proliferative Effects of an Extra-Virgin Olive Oil Extract Enriched in Ligstroside Aglycone and Oleocanthal on Human Liver Cancer Cell Lines
Source: Cancers (Basel). 2019 Oct 24;11(11):1640. doi: 10.3390/cancers11111640 (PMC6896128; doi:10.3390/cancers11111640)
Supplement: Supplementary file 1 [file cancers-11-01640-s001.zip › Figure S6.pdf]

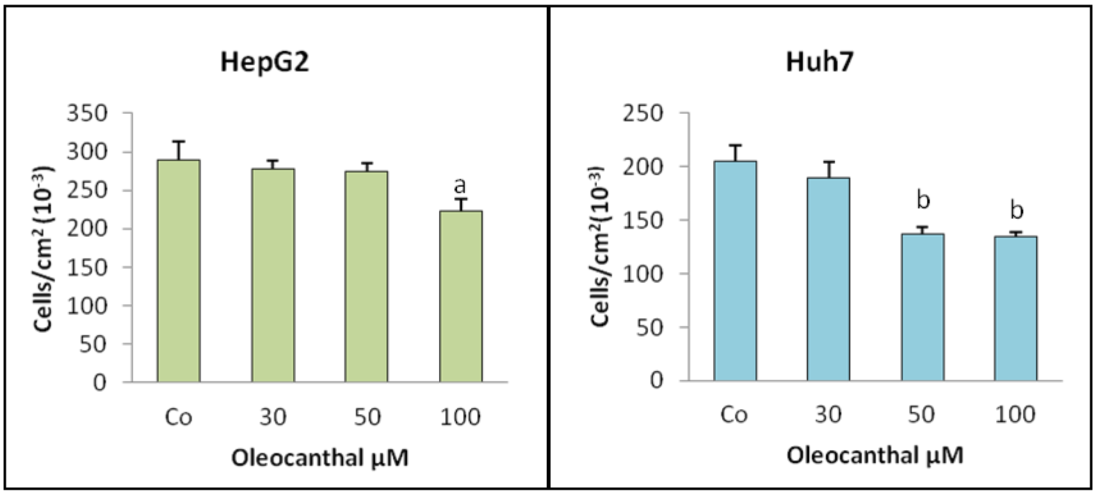

Figure S6: Growth curve of HepG2 and Huh7 cell lines incubated for 48 h with three different doses of oleocanthal. The results are representative of three experiments. Data are presented as mean ± SD. (b p<0,01 vs Co).
