# Supplementary material for: Anti-Proliferative Effects of an Extra-Virgin Olive Oil Extract Enriched in Ligstroside Aglycone and Oleocanthal on Human Liver Cancer Cell Lines
Source: Cancers (Basel). 2019 Oct 24;11(11):1640. doi: 10.3390/cancers11111640 (PMC6896128; doi:10.3390/cancers11111640)
Supplement: Supplementary file 1 [file cancers-11-01640-s001.zip › Figure S7.pdf]

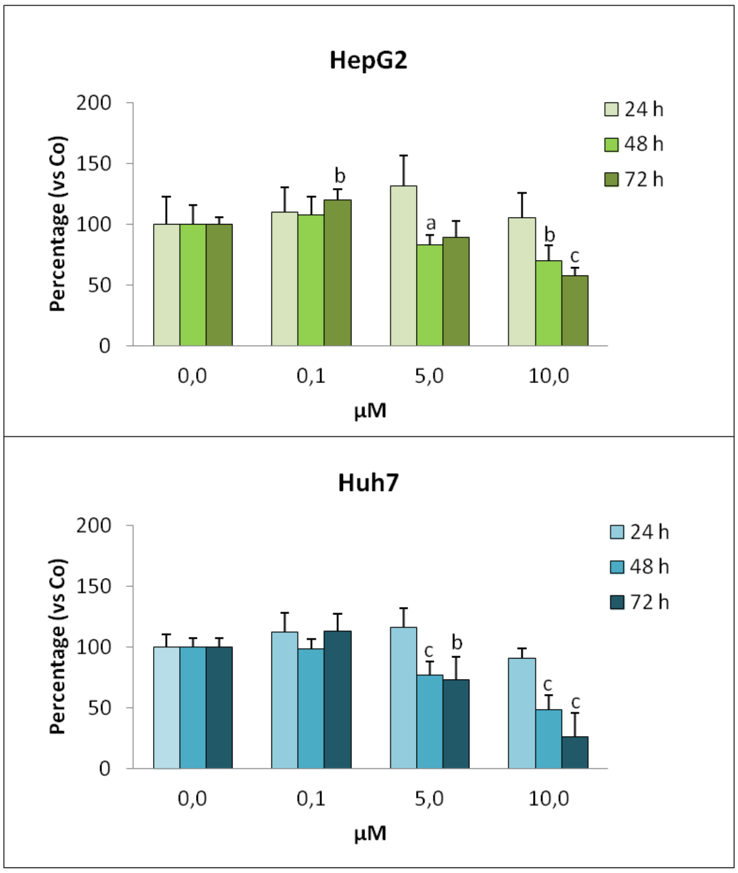

Figure S7: Dose-response experiments of HepG2 and Huh7 cell lines incubated for 24-72 h with different doses of EVOO. The indicated concentrations refer to the amount of OC present in the phenol extract. The results are representative of three experiments. Data are presented as mean ± SD. (a $p<0,05$, b $p<0,01$, c $p<0,001$ vs Co).
