# Supplementary material for: Anti-Proliferative Effects of an Extra-Virgin Olive Oil Extract Enriched in Ligstroside Aglycone and Oleocanthal on Human Liver Cancer Cell Lines
Source: Cancers (Basel). 2019 Oct 24;11(11):1640. doi: 10.3390/cancers11111640 (PMC6896128; doi:10.3390/cancers11111640)
Supplement: Supplementary file 1 [file cancers-11-01640-s001.zip › Table S1.pdf]

Table S1: Flow cytometric analysis of HepG2, Huh7 and Hep3B cell lines incubated for 24 and 48 h with different doses of the phenolic extract in the presence or absence of TNFα. The results are representative of three experiments. Data are presented as mean ± SD. (a p<0,05, b p<0,01, c p<0,001 vs Co; d p<0,05, e p<0,01).

		24 h			48 h	
**HepG2**	**G0/G1**	**S**	**G2/M**	**G0/G1**	**S**	**G2/M**
**Co**	50.71±1.44	21.53±2.67	27.76±1.24	50.86±2.00	16.13±1.01	30.90±1.24
TNFα	49.89±1.95	21.90±1.98	28.21±3.93	51.55±0.52	16.25±0.92	32.20±0.43
**EVOO1**	49.61±2.45	22.33±0.29	28.06±2.61	51.64±1.18	17.46±2.06	30.90±1.24
TNFα/EVOO1	50.27±0.67	18.94±0.50[c]	30.80±0.77[a]	56.12±2.46[a,d]	14.86±0.81	29.02±1.83
**EVOO2**	47.43±1.23[a]	15.85±0.98[a]	36.73±2.16[b]	56.33±1.44[a]	12.33±1.36[a]	31.34±2.80
TNFα/EVOO2	50.29±0.52[d]	11.53±0.46[c,e]	38.15±0.07[c]	57.05±0.90[a]	12.86±0.67[b]	30.09±1.51
**Huh7**	**G0/G1**	**S**	**G2/M**	**G0/G1**	**S**	**G2/M**
**Co**	43.63±1.71	17.00±0.39	39.36±1.79	48.06±0.19	11.17±1.04	40.77±1.22
TNFα	46.33±0.72	14.62±1.09[a]	39.08±1.53	49.31±0.94	10.71±0.26	39.98±0.98
**EVOO1**	45.63±1.07	13.29±0.85[b]	41.50±1.52	43.93±1.25[b]	12.75±1.93	43.33±2.09
TNFα/EVOO1	49.03±0.36[b,e]	11.70±0.91[c]	39.26±0.69	44.42±0.69[c]	12.91±1.38	42.67±0.71
**EVOO2**	40.70±1.10	18.65±1.45	40.66±2.28	36.92±1.44[c]	19.21±0.29[c]	43.97±1.68
TNFα/EVOO2	37.49±4.25	18.75±1.65[c]	43.76±3.01	38.02±0.49[c]	21.42±1.68[c]	40.56±1.19[d]
		**24 h**			**48 h**	
**Hep3B**	**G0/G1**	**S**	**G2/M**	**G0/G1**	**S**	**G2/M**
**Co**	45.35±0,54	38.21±1,45	17.77±2,4	44.42±6,61	37.45±1,58	18.14±5,89
TNFα	44.97±0,13	37.79±0,14	17.25±0,26	46.63±0,56	34.99±0,13	18.60±0,43
**EVOO2**	47.18±,0,93[a]	28.74±0,89[c]	24.98±0,75[a]	36.01±0,84[c]	40.92±2,47	23.07±1,64[a]
TNFα/EVOO2	43.73±2,51	29.89±0,56	26.40±1,96	30.74±0,30[d]	45.85±1,85	23.41±1,56
